# Preventive Effects of a CVD Polypill on Developing Diabetes Among Patients With Metabolic Syndrome: Results of the PolyIran-Liver Trial

**DOI:** 10.34172/aim.31839

**Published:** 2024-10-01

**Authors:** Elham Jafari, Hossein Poustchi, Abbas Mohagheghi, Maryam Sharafkhah, Masoud Khoshnia, Alireza Nateghi, Shahin Merat, Reza Malekzadeh

**Affiliations:** ^1^Liver and Pancreatobiliary Diseases Research Center, Digestive Diseases Research Institute, Tehran University of Medical Sciences, Tehran, Iran; ^2^Digestive Disease Research Center, Digestive Disease Research Institute, Tehran University of Medical Sciences, Tehran, Iran; ^3^Digestive Oncology Research Center, Digestive Disease Research Institute, Tehran University of Medical Sciences, Tehran, Iran; ^4^Department of Cardiology, Shariati Hospital, Tehran University of Medical Sciences, Tehran, Iran; ^5^Golestan Research Center of Gastroenterology and Hepatology, Golestan University of Medical Sciences, Gorgan, Iran; ^6^Research and Development Department, Alborz-Darou Pharmaceutical Co., Ghazvin, Iran

**Keywords:** Cardiovascular disease, Diabetes mellites, Fixed dose combination, Metabolic syndrome, Polypill, Primary prevention

## Abstract

**Background:**

While cardiovascular disease (CVD) polypills have demonstrated significant benefits in preventing CVD events by managing CVD risk factors and improving patient adherence, their effects on blood glucose levels, an important risk factor for CVD, remain unknown.

**Methods:**

We analyzed data from the PolyIran-Liver trial, which involved 1,508 participants aged 50 and above. Of these, 787 were randomly assigned to receive a polypill (consisting of aspirin, atorvastatin, hydrochlorothiazide, and valsartan), while 721 received usual care as the control group over a five-year period. The aim was to determine whether there were any significant differences in fasting blood sugar (FBS) levels between the two groups at baseline, middle, and end of the study. The data analysis focused on three subgroups: participants with diabetes, those with metabolic syndrome (MetS) but without diabetes, and participants without diabetes or MetS.

**Results:**

Of the total studied population, with a mean age of 59±6.7 years, 328(22%) were identified with diabetes, 371 (25%) with MetS but without diabetes, and 809 (54%) without diabetes or MetS. We observed a trend of rising FBS levels until month 30, followed by a subsequent decline at month 60. Participants in the polypill group exhibited lower FBS levels than the control group at both time points, with statistically significant differences in all three subgroups at month 30 and in the MetS-without diabetes at month 60 (mean difference: -9.3 mg/dl, 95% CI: 13.9 to -4.6).

**Conclusion:**

The polypill used in this study may have the potential to delay the onset of diabetes in patients with MetS more effectively than in the general population. However, its beneficial effects on the blood sugar levels of diabetic individuals require further investigation.

## Introduction

 Cardiovascular diseases (CVDs) are the most common cause of global mortality, placing a heavy burden on the economies and healthcare systems worldwide.^1,2^ Among several pharmacological and non-pharmacological methods for CVD prevention, the CVD polypill has gained considerable attention in recent years. Large-scale studies have confirmed its effectiveness, safety, and cost-effectiveness in both the primary and secondary prevention of CVD events.^3-6^

 A CVD polypill is a fixed-dose combination of various medications targeting different metabolic risk factors for CVD. Despite heterogeneity in the design of CVD polypill formulation, they commonly contain blood pressure-lowering drugs, lipid-reducing agents, and antiplatelet medications. Therefore, their beneficial effects on reducing CVD events by 30% to 40% are attributed to their ability to lower blood pressure, reduce serum lipid levels, prevent atherosclerosis and coagulopathies, and increase adherence.^7-10^

 Elevated blood sugar is a crucial risk factor that needs to be controlled to prevent CVDs, especially in high-risk individuals. Despite existing concerns about the role of certain components of the CVD polypill, such as statins, beta-blockers, and thiazide in increasing blood sugar levels, the impact of CVD polypills on blood sugar levels has not been thoroughly explored so far.^11-13^ On the other hand, some review publications have suggested that CVD polypills with proper combination might be efficient in preventing both CVD and diabetes, particularly in patients with metabolic syndrome (MetS).^14-16^ MetS, characterized by a cluster of metabolic abnormalities, is a key risk factor for both CVD and diabetes. Individuals with diabetes or MetS face a considerably higher risk of developing CVDs and are potential candidates for using CVD polypills.^17-20^ It is possible that CVD polypills could delay the progression of elevated blood sugar levels and developing diabetes by improving various aspects of MetS and insulin resistance, in addition to raising adherence. This would present a simpler and more cost-effective method for preventing diabetes and heart disease among these high-risk populations.^21-24^

 Determining the effects of CVD polypill on these patients’ blood sugar is crucial as it provides essential information for optimizing its formulation, boosting efficacy, and improving clinical guidelines. Therefore, we assessed the impact of the CVD polypill utilized in the PolyIran-Liver trial on fasting blood sugar (FBS) levels over five years, taking into account pre-existing diabetes, MetS, or the absence of these conditions at the initiation of the trial.

## Materials and Methods

 This study was based on data obtained from the PolyIran-Liver study, an open-labeled randomized controlled trial embedded within the Golestan Cohort Study (GCS). The GCS is a large-scale prospective cohort involving 50 000 individuals aged 40 to 75 from the general population of the Golestan province, located in northeastern Iran.^25^ In the PolyIran-Liver study, 2400 individuals aged 50 and above were randomly selected from the urban population of the GCS using a computer-generated list, with a 50:50 sex ratio. These individuals were then randomly assigned to either the intervention or control groups. Participants with active hepatitis, viral hepatitis, and contraindications to the components of the polypill or those who did not provide consent to the trial were excluded from the study. Ultimately, 1508 participants (787 in the intervention group and 721 in the control group) met the eligibility criteria, consented to participate in the trial, and were enrolled for further assessments. Participants in the intervention group were prescribed a daily polypill consisting of 81 mg aspirin, 12.5 mg hydrochlorothiazide, 20 mg atorvastatin, and 40 mg valsartan for five years.

 Participants in the intervention group who already took medications included in the polypill underwent dosage adjustments. Information regarding the management of these participants was previously published in the protocol.^26^ The participants in both groups were monitored every six months for five years. During each visit, their body composition and blood pressure were measured, and they filled out a questionnaire about adverse events or additional medications taken. Additionally, a pill count was conducted during each follow-up visit to assess adherence to the prescribed polypill. In case of any diagnosed disorder in the control group, they were referred to a physician for further evaluation and received conventional medical care, including statins, glucose-lowering agents, or antihypertensive medications when necessary.^26^

###  Variable and Definitions

 Data from the PolyIran-Liver study were stratified into three categories: pre-existing diabetes, existing MetS without diabetes, and the absence of both conditions at the beginning of the trial. This stratification allowed for a more detailed analysis of the study results within these specific subgroups. Diabetes was defined by either self-reporting of pre-existing diabetes accompanied by taking antihyperglycemic medication or having fasting plasma glucose > 125 mg/dL, or Hb A1C > 6.5% at the start of the study. MetS was defined according to the Third Report of the National Cholesterol Education Program (NCEP ATP III), requiring at least three of the following five metabolic abnormalities: (1) elevated fasting glucose ( ≥ 100 mg/dL) or specific drug treatment, (2) elevated blood pressure (systolic ≥ 130 mmHg or diastolic ≥ 85 mmHg or antihypertensive drug treatment), (3) reduced HDL cholesterol ( < 40 mg/dL in men or < 50 mg/dL in women or on specific drug treatment), (4) elevated triglycerides ( ≥ 150 mg/dL or on specific drug treatment), and (5) elevated waist circumference( ≥ 90 cm based on other published studies).^27,28^ The subgroup of MetS without diabetes was defined by excluding participants with diabetes from the MetS participants.

###  Outcome 

 The outcome measured was the occurrence of statistically significant differences in the FBS changes between the polypill and control groups within each of the three subgroups at the middle and end of the study.

###  Statistical Methods

 The sample size justification for the PolyIran-Liver has been previously described.^26^ In this study, we determined that the sample provided over 90% power to detect a minimum difference of 5 mg/dL in FBS between the intervention and control groups, assuming that the highest observed standard deviation in baseline changes was 10 mg/dL. Baseline characteristics were compared between the study groups using independent t-tests for continuous variables and chi-squared tests for the categorical variables.

 Changes in FBS levels were analyzed using a generalized linear model with normal distribution for random error and identity link function. To control for the potential effects of antidiabetic agents, metabolic dysregulation, and glucose homeostasis on our findings, we analyzed FBS changes in three strata according to pre-existing diabetes, existing MetS without diabetes, and the absence of both conditions at the beginning of the trial. Moreover, further adjustments were made in three models to eliminate the effects of the other potential confounder factors for each stratum: (1) adjusting for baseline FBS values, (2) further adjusting for age, sex, body mass index (BMI), and waist circumference, and (3) further adjustment for baseline CVD medication (lipid-lowering drugs, antihypertensives). All analyses were conducted by Stata software (version 12), and a *P* value of less than 0.05 was considered statistically significant.

## Ethical Considerations

 Informed consent was obtained from all participants. The PolyIran-Liver study protocol was approved by the institutional review board of the Digestive Diseases Research Institute of the Tehran University of Medical Sciences and ethics committees from the Tehran University of Medical Science and the Ministry of Health and Medical Education in Iran. The trial was registered at ClinicalTrials.gov, ID: NCT01245608.^26^

## Results

 Out of 1508 participants who consented to the PolyIran trial, 787 were in the polypill group and 721 in the control group. Participants in both groups had similar mean ages (58.5 ± 6.3 in the polypill group and 59.6 ± 6.9 in the control groups). Males comprised 50% of the participants, with a slightly higher rate in the control group. The proportion of participants with pre-existing diabetes at baseline was similar between both groups (22.2% in the polypill group and 21.2% in the control group). Moreover, participants with MetS but without diabetes (Non-diabetic MetS) showed an equal proportion (25%) in both groups (Table 1).

**Table 1:** Baseline Characteristics of the Participants

**Characteristics**	**Polypill (n=787)**	**Control (n=721)**	***P*** ** value**
Age, years (Mean ± SD)	58.5 ± 6.3	59.6 ± 6.9	0.062
Male, N (%)	396 (50)	376 (53)	0.477
Pre-existing CVD, N (%)	144 (18.3)	97 (13.5)	0.072
Pre-existing HTN, N (%)	414 (52.6)	404 (56.1)	0.502
Pre-existing DM, N (%)	174 (22.1)	152 (21.1)	0.633
Nondiabetic MetS, N (%)	198 (25.1)	173 (24.9)	0.580

*Note.* SD: Standard deviation; CVD: Cardiovascular disease; HTN: Hypertension; DM: Diabetes mellitus: MetS: Metabolic syndrome.

 We identified 328 (22%) participants with diabetes, 371(25%) with MetS but without diabetes, and 809 (54%) without diabetes or MetS. As seen in Table 2, the mean ages between these three subgroups and between the polypill and control groups were almost equal. The proportion of men in the diabetic group was slightly lower than in the other two groups. As expected, the metabolic measurements were higher in the diabetic and MetS subgroups than in those without these conditions, except for creatinine and blood urea nitrogen (BUN), which were equal across all three subgroups.

**Table 2:** Baseline Characteristics of the Three Analyzed Subgroups

**Characteristics**	**Diabetes (n=328)**		**MetS and Non-diabetes (n=371)**		**Non-MetS and Non-Diabetes (n=809)**	
	**Polypill (n=175**	**Control (n=153)**	**Poly-pill (n=198)**	**Control (n=173)**	**Poly-pill (n=414)**	**Control (n=395)**
Male gender, n (%)	71 (41)	67 (44)	93 (47)	94 (54.3)	232 (56.0)	215 (54.4)
Age > 65 years, n (%)	24 (13.7)	32 (20.9)	29 (16.8)	32 (18.7)	61 (15.8)	97(24.6)
Age, mean (SD)	59.3 (6.6)	59.3 (6.7)	58.7 (6.3)	59.0 (6.4)	58.2 (6.2)	59.6 (7.3)
BMI, mean (SD)	30.2 (4.7)	29.9 (5.0)	29.9 (4.1)	29.4 (3.9)	27.2 (5.0)	27.2 (5.0)
Weight, mean (SD)	76.9 (12.8)	77.2 (13.9)	77.4 (10.9)	78.2 (12.5)	71.8 (13.5)	71.3 (13.8)
WC, mean (SD)	104.9 (10.8)	104.8 (11.2)	103.8 (9.3)	102.9 (9.7)	96.3 (12.8)	96.4 (12.3)
SBP, mean (SD)	137.6 (22.5)	138.2 (18.7)	137.7 (19.7)	139.8 (19.9)	127.0 (21.5)	132.1 (21.7)
DBP, mean (SD)	79.2 (9.3)	81.9 (9.6)	81.8 (9.6)	86.6 (11.4)	77.7 (10.1)	81.3 (10.3)
FBS, mean (SD)	173.4 (61.5)	170.7 (61.5)	98.8 (10.9)	95.2 (10.6)	94.2 (11.3)	91.4(9.4)
HBA1c, mean (SD)	8.1 (1.6)	7.1 (2.8)	6.1 (0.6)	5.4 (1.8)	6.1 (0.9)	4.2(1.9)
Insulin, median (IQR)	12.4 (8.5)	9.4 (8.2)	12.9 (9.7)	10.0 (5.8)	8.3 (6.9)	7.5 (5.9)
HOMA-IR, median (IQR)	5.2 (4.5)	4.0 (3.7)	3.2 (2.4)	2.3 (1.6)	1.9 (1.6)	1.6 (1.4)
TG, median (IQR)	173.0 (120.0)	153.0 (107.5)	192.0 (75.5)	179.0 (88.0)	109.0 (51.0)	99.5 (50.0)
TC, mean (SD)	223.4 (46.8)	209.2 (45.4)	224.8 (44.5)	211.5 (40.9)	217.9 (37.4)	205.6 (37.9)
HDL, mean (SD)	57.6 (14.5)	56.2 (14.1)	53.1 (13.0)	50.2 (11.6)	64.9 (14.4)	62.8 (14.1)
LDL, mean (SD)	128.1 (34.8)	117.6 (35.8)	130.9 (37.6)	122.6 (34.7)	129.7 (31.4)	121.6 (31.1)
BUN, mean (SD)	30.2 (8.1)	29.5 (7.8)	29.2 (7.2)	28.3 (6.6)	29.6 (8.9)	29.1 (6.7)
Cr, mg/dL, mean (SD)	1.2 (0.3)	1.1 (0.2)	1.2(0.2)	1.1(0.2)	1.2 (0.2)	1.1 (0.2)

Note. SD: Standard deviation; BMI: Body mass index; WC: Waist circumference; SBP: Systolic blood pressure; DBP: Diastolic blood pressure; FBS: Fasting blood sugar; HBA1c: Hemoglobin A1c; HOMA-IR: Homeostatic model assessment for insulin; TG: Triglyceride; TC: Total cholesterol; HDL: High-density lipoprotein cholesterol; LDL: Low- density lipoprotein cholesterol; BUN: Blood urea nitrogen; Cr: Creatinine.

 The median adherence to polypill among the intervention group was 85% (IQR 60–94%) and was similar across all three subgroups of the study (*P *value = 0.942).

 When comparing the effects of polypill on the FBS levels with usual care among diabetic participants, we noticed an upward trend in the FBS levels from the baseline in both the polypill and control groups at month 30. However, the polypill group showed a lower increase, with a mean difference of -19.8 mg/dL (95% CI -38.4 to -1.2), than the control group. At month 60, both groups experienced a reduction. The polypill group declined below baseline, while the control group remained slightly above it, with the mean difference of -6.6 mg/dL (95% CI -28.0 to -14.8), as depicted in Table 3.

**Table 3:** Changes in Mean FBS Levels by Study Arms at 30 and 60 Months

	**Polypill**	**Control **	**Mean Difference**	***P*** ** Value**	***P*** ** Value (Model I)**	***P*** ** Value (Model II)**	***P*** ** Value (Model III)**
Participants with diabetes (n = 328)							
Month 30	127 (72%)	117 (76%)	-	-	-	-	-
Month 60	126 (72%)	76 (49%)	-	-	-	-	-
Change in FBS levels from baseline, mean (95% CI)							
Month 30	11.6 (-0.4 to 23.6)	31.5 (17.2 to 46.0)	-19.8 (-38.4 to -1.2)	0.037	0.047	0.081	0.129
Month 60	-4.8 (-18.9 to 9.3)	1.8 (-14.9 to 18.5)	-6.6 (-28.0 to 14.8)	0.543	0.957	0.901	0.763
Participants with MetS but without diabetes (n = 371)							
Month 30	152(76%)	143(82%)	-	-	-	-	-
Month 60	144(72%)	101(58%)	-	-	-	-	-
Change in FBS levels from baseline, mean (95% CI)							
Month 30	5.5 (2.9 to 8.1)	14.1 (10.8 to 17.4)	-8.6 (-12.7 to -4.3)	0.001	0.001	0.001	0.001
Month 60	2.8 (0.2 to 5.3)	12.2 (8.0 to 16.4)	-9.3 (-13.9 to -4.6)	0.001	0.001	0.001	0.001
Participants without MetS /diabetes (n = 809)							
Month 30	339 (81%)	324 (82%)	-	-	-	-	-
Month 60	327(78%)	223 (56%)	-	-	-	-	-
Change in FBS levels from baseline, mean (95% CI)							
Month 30	6.4 (4.8 to 8.0)	10.2 (8.8 to 11.6)	-3.8 (-6.4 to -1.2)	0.004	0.024	0.036	0.041
Month 60	3.9 (2.0 to 5.9)	5.7 (3.0 to 8.4)	-1.8 (-4.8 to 1.1)	0.223	0.551	0.335	0.225

*Note. *MetS: Metabolic syndrome; FBS: Fasting blood sugar; CI: Confidence interval. Model I: Models were adjusted for the baseline value of the outcome. Model II: Models were further adjusted for age, sex, body mass index, and waist circumference. Model III: Models were further adjusted for non-trial cardiovascular disease medication.

 In participants with MetS but without diabetes, the polypill group exhibited a lower increase in mean FBS levels compared with the control group at month 30, with a mean difference of -8.6 mg/dL (95% CI -12.7 to - 4.3). By month 60, FBS levels decreased to around baseline levels but still remained lower than those in the control group, with a mean difference of -9.3 mg/dL (95% CI -13.9 to - 4.6), as illustrated in Table 3.

 Among participants without Mets or diabetes, the polypill group showed a smaller increase in FBS levels compared to the control group, with a mean difference of -3.4 mg/dL (95% CI -6.4 to -1.2). By month 60, FBS levels decreased in both groups, and the changes in the two groups had become closer, with a mean difference of -1.8 mg/dL (95% CI -4.8 to 1.1), as depicted in Table 3. Furthermore, Table 3 indicates that all results were adjusted for potential confounding variables, but no significant changes were observed.

## Discussion

 In this pragmatic randomized controlled trial, we observed that polypill consumption in all three subgroups resulted in a statistically significant lower increase in FBS levels compared to the control group at month 30 and a higher decrease at month 60; however, the latter change was statistically significant only in participants with MetS but without diabetes. This novel finding suggests that the utilized CVD polypill may have the potential to delay the onset of diabetes, with greater efficacy in participants with MetS and could be considered a preventive method for diabetes with CVD events in these high-risk patients. These findings agree with previous review studies that demonstrated the beneficial effects of CVD polypills with the right combination in the primary prevention of CVD and diabetes in MetS patients.^14^ Recently, a meta-analysis suggested that using blood pressure-lowering drugs, especially angiotensin-converting enzyme inhibitors and angiotensin II receptor blockers for five years, reduces the risk of new-onset type 2 diabetes by 16%, while the use of β-blockers and thiazide diuretics increased this risk by 1.20% and 1.02%, respectively. No material effect was found for calcium channel blockers.^29^ Furthermore, another clinical trial indicated that using Renin-Angiotensin System Blockade combined with statin Improves endothelial function in diabetic patients.^30^

 In our previous study, we used data from the PolyIran-Liver trial to assess polypill’s effects on preventing CVD events and mortality in patients with metabolic-associated fatty liver disease (MAFLD). We observed that the used polypill has a more significant preventive effect in participants with MAFLD compared to the general population, which could not be attributed to increased adherence since adherence levels were similar in both study groups.^31^

 Given the equal adherence rates among the polypill arms of the studied subgroups in the current study, the superior protective effect of the polypill used in the PolyIran-Liver trial may be attributed to the inclusion of Valsartan, an antihypertensive medication, in classes of angiotensin-converting enzyme inhibitors, along with satins and aspirin, which are anti-inflammatory agents. This combination in the CVD polypill used in our studies could provide a synergic inhibitory effect on inflammatory pathways and metabolic dysregulation.

 Our data showed that the polypill significantly reduced FBS levels in participants with diabetes compared to the control group during the first half of the study. However, the smaller difference in FBS levels between the polypill and control groups at month 60 may be attributed to the use of conventional medical treatments for diabetes in the diabetic participants, potentially underestimating the polypill’s effect on reducing FBS levels. In addition, higher dropout rates in long-term follow-up may have resulted in reduced sample size and study power, further diluting the polypill’s impact in this subgroup.

 Furthermore, the results of this study showed that polypill intake could partially inhibit rising FBS levels in the general population after two and a half years; however, this effect was not evident by the end of five years. Confirming these findings in future studies would be valuable, as it would provide new access to more practical pharmacological approaches with high adherence for delaying metabolic dysregulation and preventing metabolic diseases in high-risk populations, particularly concerning genetics and age.

 Our study had several strengths. First, our data were obtained from a randomized controlled trial design, a gold research standard that helps minimize bias. Second, subgroup analysis of different groups (people with and without diabetes and those with and without MetS) provides more detailed information about the polypill’s effects on various populations, which were unknown. Third, following participants for 60 months allowed us to observe changes in FBS levels over a long time. Finally, it was the first investigation in this field, showing that the polypill not only prevents cardiac events but may also help prevent diabetes and MetS,

 As previously stated, an important limitation of this study was the use of conventional medical treatments in the control group and the lack of a placebo, which may have underestimated the polypill’s potential to inhibit rising glucose levels. However, we analyzed people with diabetes separately to eliminate the effect of metformin and other antidiabetics. Another limitation was the lack of HbA1C and insulin levels in long-term assessments. Since the PolyIran-Liver trial was primarily designed to observe changes in CVD events rather than risk factors, we only had FBS measurements at months 30 and 60. Thus, we could not detect new incidences of diabetes and evaluate the effects of a polypill more accurately in this case. Finally, since the participants in this study were over 50, the natural increase in FBS levels associated with aging may have undervalued the polypill’s effects on blood sugar control even though the polypill group was superior to the control group over five years. Therefore, we recommend more large-scale studies with a more comprehensive age range to support our results, generalize them to a broader population, and provide more power to detect significant differences between groups.

## Conclusion

 This study represents the first attempt to address the knowledge gap and previous doubts regarding the effects of CVD polypills on blood sugar levels. It indicated that the CVD polypill used in the PolyIran-Liver trial has the potential to improve FBS levels compared to standard care and may even delay the onset of diabetes in participants with MetS. Additionally, the findings highlighted that the impact of CVD polypills on FBS levels varied substantially depending on participants’ metabolic conditions at baseline. These novel insights provide new evidence and a foundation for further comprehensive research in this field to optimize CVD polypill formulations for the prevention of CVD and diabetes, particularly in individuals with MetS who are at a greater risk of developing diabetes and CVD events.
